# HER2 c-Terminal Fragments Are Expressed via Internal Translation of the HER2 mRNA

**DOI:** 10.3390/ijms23179549

**Published:** 2022-08-23

**Authors:** Jack D. Godfrey, Daniel Hejazi, Xiaofei Du, Cenfu Wei, Eshaan Rao, Christopher M. Gomez

**Affiliations:** Department of Neurology, University of Chicago, Chicago, IL 60637, USA

**Keywords:** HER2, IRES, translation

## Abstract

The HER2/neu signaling pathway is one of the most frequently mutated in human cancer. Although therapeutics targeting this pathway have good efficacy, cancer cells frequently develop resistance. The HER2 gene encodes the full-length HER2 protein, as well as smaller c-terminal fragments (CTFs), which have been shown to be a cause of resistance. Here, we show that HER2 CTFs, exclusive from the full-length HER2 protein, are generated via internal translation of the full-length HER2 mRNA and identify regions which are required for this mechanism to occur. These regions of the HER2 mRNA may present novel sites for therapeutic intervention via small molecules or antisense oligonucleotides (ASOs).

## 1. Introduction

Human epidermal growth factor receptor 2 (HER2/neu) is encoded in the *ERBB2* gene and is a member of the epidermal growth factor receptor (EGFR) family of membrane-bound receptor tyrosine kinases. This family consists of four proteins, each with an intracellular domain, a transmembrane domain and a large extracellular ligand-binding domain, although in the case of HER2, no ligands have been identified to date. Downstream signaling by HER2 is primarily through the PI3K/AKT signaling pathway [1] and requires dimerization with other ligand-bound EGFR family members [2]. *ERBB2* is found to be amplified in a number of tumor types of epithelial origin including lung, gastric and bladder cancers [3] and is a major cause of breast cancers, accounting for 15% of cases [4] where amplification drives aggressive and invasive tumor formation.

The major protein product of the *HER2* mRNA is the 180KDa HER2 transmembrane cell surface receptor. However, nuclear accumulation of the full HER2 protein has been observed in breast cancer [5] and has been shown to bind to the promoters of a subset of genes [6], including COX2 where it stimulates transcription [7]. In addition to full-length HER2, smaller proteins with estimated molecular weights between 75-100KDa have also been identified, which are composed of the c-terminus of HER2.

The CTFs known as HER2p95 are generated via proteolytic cleavage of full-length HER2 by alpha-secretase [8]. This results in peptides starting at A648 and E645 which contain both a nuclear localization signal (NLS) and a transmembrane domain (TM), with HER2-A648 being the major cleavage product [9,10]. A second cleavage event mediated by gamma-secretase produces a peptide beginning at K676 that lacks the TM but contains the NLS, enabling nuclear translocation [11]. In addition to these cleavage events, there is data suggesting that a number of CTFs are generated by internal translation of the *HER2* mRNA. These events result in peptides that begin at either M611, which contains the TM and NLS, or at M687 [12] which lacks both, although they have still been observed in the nucleus [11]. Importantly, HER2 CTFs that contain the TM, HER2-A648 and, to a much greater extent, HER2-M611, retain downstream tyrosine kinase signaling activity as measured by phosphorylation of MAPK, Scr, Akt and PLCγ. CTFs lacking the TM, HER2-M687 and HER2-K676, do not appear to have tyrosine kinase activity [11]. Additionally, ectopic expression of M611 leads to a significant change in gene expression, greater even than HER2 overexpression in MCF7 cells, whereas HER2-M687 and HER2-K676 have no significant effect on gene expression [11]. Critically, transgenic mice expressing HER2-M611 have a greater number of tumors with a larger volume and more aggressive growth compared to mice overexpressing full-length HER2, whereas HER2-M687 overexpression does not result in tumor formation, with the mammary glands being comparable to control mice [11]. Other studies show that HER2 CTFs drive aggressive tumor formation in mice and that the migration of HER2 M611-expressing cells is dependent on cortactin phosphorylation [13].

In human tumors, HER2 CTFs are sufficient to drive metastasis and, in some cases, are the predominant HER2 proteins [14]. HER2 CTF expression has also been shown to downregulate the estrogen receptor (ER), leading to reduced efficacy of ER-targeting drugs [15]. Expression of HER2 CTFs can result in resistance to conventional HER2-targeting antibody-based therapeutics [16], with patients with a high HER2 CTF to HER2 ratio having poor prognostic outcomes [17]. Importantly, although the Scaltriti treatment with trastuzumab has limited efficacy in patients who express HER2 CTFs, the tyrosine kinase inhibitor (TKI) lapatinib has significant clinical impact [18]. Although TKIs may be effective therapeutics in the treatment of tyrosine kinase-driven cancers, they are frequently defeated by secondary mutations known as gatekeeper mutations. The gatekeeper mutant in HER2 is the T798M mutation, which desensitizes tumors to lapatinib treatment [19]. Given this, understanding the mechanism of HER2 CTF generation will allow for the development of therapeutics directed at inhibiting the expression of HER2 CTF, ideally while sparing full-length HER2 expression and allowing efficacious treatment using trastuzumab. 

The process of eukaryotic cap-dependent translation initiation is a complex procedure involving interplay between multiple protein complexes called eukaryotic initiation factors and the ribosome. We overview the topic in brief, but for a detailed review please see [20]. The initial process of initiation is the binding of eIF4F, a complex composed of eIF4E which interacts with the cap structure, eIF4A, which has helicase activity and eIF4G, which is a scaffold complex which interacts with eIF3 and polyA-binding protein (PABP). The next step involves the 43S pre-initiation complex (43S PIC), comprising eIF1, eIF1A, eIF3, the 40S ribosomal subunit and eIF2 loaded with an initiator methionine tRNA, which interacts with eIF4F to produce the 48S complex. The 48S complex scans from the cap structure towards the start codon where the 60S ribosomal subunit interacts with the 40S ribosomal subunit to form the translationally competent 80S ribosome, and translation is initiated.

Aside from canonical, cap-dependent mechanisms, cap-independent initiation may also occur, which typically relies on RNA elements called internal ribosome entry sites (IRESs). IRES elements are found in a number of viruses where they can be separated into four groups: group 1 IRESs require no eukaryotic initiation factors; group 2 IRESs require eIF2 and eIF3; group 3 requires eIF2, eIF3, eIF4A, eIF4B and eIF4G; and group 4 requires eIF2, eIF3, eIF4A and eIF4G (reviewed [21]).

IRES elements have also been identified in cellular mRNAs, typically in the 5′ untranslated region (UTR) (reviewed in [22]) which can facilitate translation where cap-dependent translation is inhibited. However, some are found downstream of the canonical start codon where they produce truncated proteins (reviewed in [23].

Here, we present data suggesting that HER2 can be added to the list of mammalian mRNAs with IRES elements. We show that HER2 CTFs are generated via internal translation of the full-length *HER2* mRNA and identify regions which are required for HER2 CTF biogenesis.

## 2. Results

### 2.1. HER2 CTFs Are Expressed from the Full-Length HER2 mRNA

**Figure 1:**
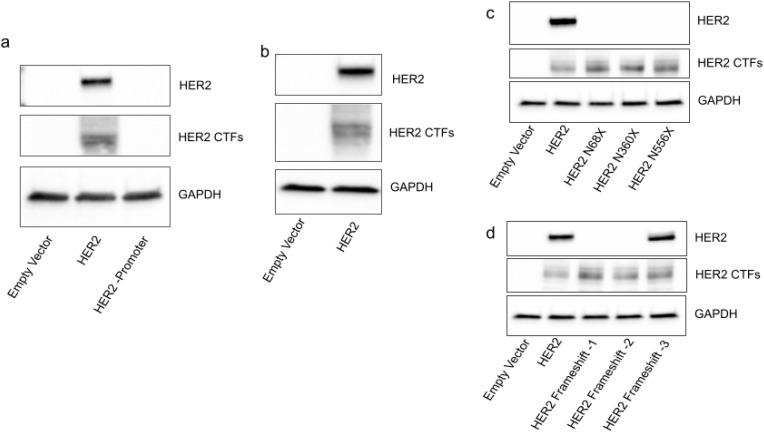
HER2M611 is expressed from the full-length HER2 mRNA. (**a**) Protein expression of cells transfected with a plasmid encoding FLAG-tagged wild-type HER2 with or without a CMV promoter, probed with an anti-FLAG primary antibody. (**b**) Protein expression from an in vitro-transcribed mRNAs encoding FLAG-tagged HER2 probed with an anti-FLAG primary antibody. (**c**) Protein expression from in vitro-transcribed mRNAs encoding FLAG-tagged HER2 with the AAU codon encoding N68, N360 and N556 mutated to stop codons, probed with an anti-FLAG primary antibody. (**d**) Protein expression from in vitro-transcribed mRNAs encoding FLAG-tagged HER2 with single nucleotide deletions to shift the frame, probed with an anti-FLAG primary antibody.

Given that HER2 CTFs are able to drive aggressive tumor formation, it is vital that we discover how they are generated to be able to specifically inhibit their expression. The first step was to interrogate the possibility of a cryptic promoter in the *HER2* gene which could be responsible for the expression of HER2 CTFs. To achieve this, equal amounts of plasmids encoding c-terminally FLAG-tagged HER2 either with or without a mammalian promoter were transfected into cells. The protein from the HER2 plasmid which had a mammalian promoter shows distinct banding patterns, with full-length HER2 running at 180 KDa, designated here as HER2, and a group of at least three bands running between 80–100 KDa, designated here as HER2 CTFs (Figure 1a), which is in line with previous studies investigating HER2 c-terminal proteins [12]. Importantly, the construct which lacks a mammalian promoter does not express either full-length HER2 or HER2 CTFs (Figure 1a). The empty vector lane proves that the detected proteins are encoded on the transfected plasmid and not endogenous proteins which cross-react with the FLAG antibody. 

The second step was to prove that HER2 CTFs are not generated due to a splicing event. This was achieved using transfection of an in vitro-transcribed mRNA encoding c-terminally FLAG-tagged HER2. The stringency of the T7 polymerase for the T7 promoter ensures that only full-length *HER2* mRNA is generated in the in vitro transcription reaction. Here, we show that this full-length mRNA is able to express both full-length HER2 and HER2 CTFs (Figure 1b).

To investigate whether HER2 CTFs are generated as cleavage products of the full-length HER2 protein, we introduced premature stop codons upstream of the largest of the HER2 CTF start sites (HER2-M611). The AAU codons encoding asparagine residues at N68, N360 and N556 were mutated to stop codons. Transfection of in vitro-transcribed mRNAs encoding these mutants into HEK293 cells shows that the full-length HER2 protein expression was completely ablated, whereas the c-terminal HER2 expression was unchanged (Figure 1c). As a confirmation, nucleotides 818, 819 and 820 were sequentially removed to generate frame shifts which should ablate HER2 protein expression. Removal of either nucleotide 818 alone or nucleotides 818 and 819 completely ablates HER2 expression while having no impact on HER2 CTF expression (Figure 1d). Importantly, when all three nucleotides are removed and the frame is reset, HER2 expression is reinstated (Figure 1d), again with no change in HER2 CTF expression. This shows that the HER2 CTFs are not all generated as cleavage products of the full-length HER2 protein.

The finding that HER2 and HER2 CTFs are generated from the same full-length mRNA that generates HER2 could be explained by either of two alternate possibilities: (1) HER2 CTFs might be generated via scanning from the cap down to the internal AUG codons or (2) HER2 CTFs might be generated via an internal initiation event. We interrogated this by introducing a hairpin with a −ΔG of −50, known to significantly reduce cap-dependent translation [24], with 28 nt downstream of the T7 site in the plasmid containing the *HER2* ORF. Both the FLAG-tagged HER2 construct and the FLAG-tagged HER2 construct containing the hairpin were in vitro-transcribed with either a 5′7-methyl-G-cap or an A-cap. As eIF4E specifically interacts with a 5′7-methyl G cap [20], the A-capped transcripts are not able to interact with eIF4E and, therefore, it is refractory to cap-dependent translation.

**Figure 2:**
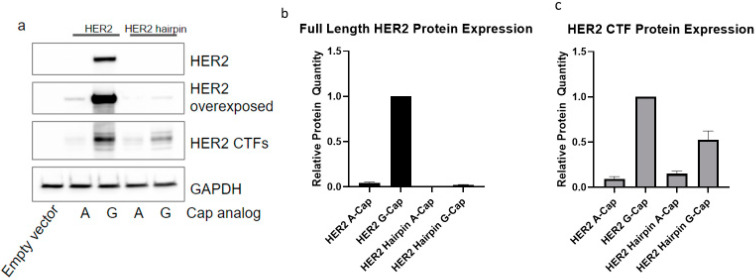
HER2 CTFs do not require a functional 5′7-methyl guanosine cap structure. (**a**) Protein expression from cells transfected with in vitro-transcribed mRNAs encoding FLAG-tagged wild-type HER2 or HER2 with a 5′ stable hairpin structure and with either functional 7-methyl-G-cap or non-functional A-caps, probed with an anti-FLAG primary antibody. (**b**) Relative protein quantification of the full-length HER2 proteins with reported values being relative to the HER2 G-capped lane. (**c**) Relative quantification of the HER2 CTF bands with reported values being relative to the CTFs in the HER2 G-capped lane. For (**b**,**c**), reported values are the mean of three experimental repeats with the error bars representing the standard deviation of replicates.

The A-capped HER2 mRNA significantly reduced expression of full-length HER2 and HER2 CTF compared to the G-capped mRNA (Figure 2a). Interestingly, the G-capped hairpin mRNA was associated with a large reduction in HER2 expression, but only mildly reduced HER2 CTF expression to around 50% of that expressed from the HER2 mRNAs without a hairpin (Figure 2b,c). Of real interest is that the A-capped hairpin mRNA has no observable expression of HER2, but expresses HER2 CTFs at roughly the same level as the A-capped HER2 mRNA (Figure 2b,c). Although HER2 CTF expression is dramatically reduced when generated from A-capped mRNAs compared to G-capped mRNAs, it is still expressed when cap-dependent translation is inhibited by a combination of a 5′ hairpin and a non-functional A-cap (Figure 2b,c).

These data suggest that HER2 CTFs are expressed most efficiently when there is a functional cap structure at the 5′ of the transcript, but also that even without a functional cap, the CTFs can be generated. Our findings also show that a hairpin structure, which likely stops a scanning ribosome, is sufficient to inhibit HER2 expression. The loss of HER2 expression from the hairpin-containing transcript has little impact on HER2 CTF expression, suggesting that the HER2 CTFs are not generated by ribosome scanning from the cap to AUGs, which encode methionines where the HER2 CTFs begin. Collectively, these findings suggest that HER2 CTFs are generated by internal initiation of the HER2 mRNA.

### 2.2. Translation of HER2 CTFs Are Initiated at a Cluster of Internal Methionine Codons

**Figure 3:**
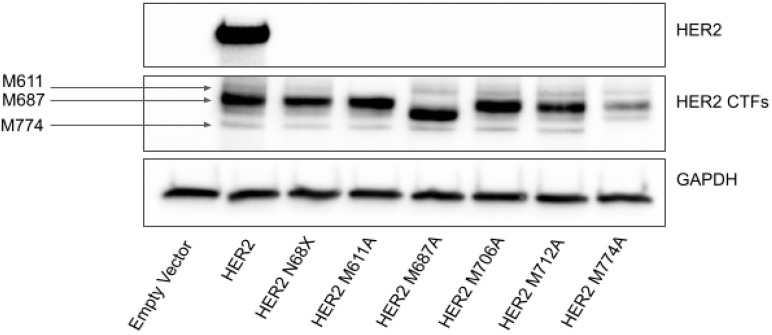
The AUG codons encoding M611, M687 and M774 are functional start codons for HER2 CTFs. Protein expression from in vitro-transcribed mRNAs encoding FLAG-tagged HER2 with endogenous methionine codons (M611, M687, M706, M712 and M774) mutated to alanine and probed with an anti-FLAG antibody.

To further test the possibility that HER2 CTFs are generated via internal translation, we sought to identify the functional start sites of the HER2 CTFs. Previous studies had shown that a cluster of methionine codons within the *HER2* mRNA are the N-terminal codons for the HER2 CTFs [12]. To identify the N-terminal codons of the HER2 CTFs, we introduced mutations to the AUG codons encoding methionine residues that had been previously investigated, as well as an additional AUG codon encoding M774. All mutations were generated in a construct bearing the N68X premature stop mutation to ensure that all of the CTFs we were interrogating were generated by the internal translation mechanism, rather than by cleavage of the full-length HER2 protein. Here, we show that, in line with previous studies, the AUG codons encoding M611 and M687 are functional start codons. Expression of protein products starting from the AUG codons encoding M706 and M712 was not observable using this method. We also show that an additional AUG encoding M774, which was not previously identified, is also functional and responsible for the smallest of the HER2 CTFs (Figure 3).

There is a clear difference in expression of the three distinct HER2 CTFs that we have identified. Although the CTFs starting at the AUG encoding M611 and M774 have a similar abundance, the major protein isoform is the CTF which initiates at the AUG encoding codon M687. To try to understand this, we utilized an informatic approach for identifying initiation sites in mRNAs using their Kozak context as well as their global sequence information [25]. This software identified 74 AUG codons in the *HER2* transcript and predicted that 33 (45%) were potentially true initiation sites and 26 were in-frame of the full-length HER2 start codon (Table S1). The AUG encoding the start codon for full-length HER2 has the highest score for prediction of a true initiation site (0.819). Interestingly, the AUG encoding the start codon for the HER2 CTF starting at M611 has a high score (0.744) and that at M687 has a significantly lower score (0.534), whereas that of M774 is not predicted to be a functional start codon and has a lower score (0.450). These scores are not sufficient to explain the difference in abundance of the HER2 CTFs, suggesting that factors other than the Kozak context, potentially protein stability, are responsible for the high expression of the HER2 CTF that starts at codon 687.

**Figure 4:**
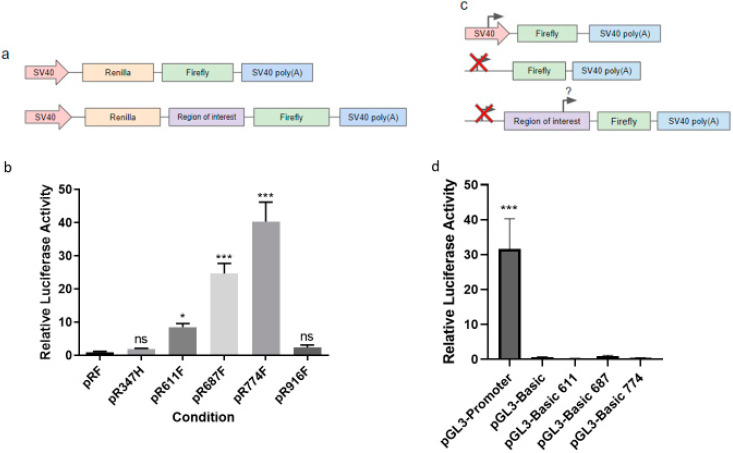
RNA fragments upstream of the HER2M611 start sites are able to facilitate internal translation. (**a**) Schematic of bicistronic reporter constructs. (**b**) Relative luciferase activity of bicistronic luciferase reporters containing 1000 bp fragments upstream of the AUG codon encoding M611, M687 and M774, as well as two control constructs containing 1000 bp fragments of HER2 mRNA upstream of the AUG codons encoding M347 and M916. Data are relative to a construct containing no insert, pRF. (**c**) Schematic of monocistronic luciferase reporter constructs. (**d**) Relative luciferase activity from monocistronic luciferase reporters containing the 1000 bp regions upstream of the AUG codons encoding M611, M687 and M774 but lacking mammalian promoters. Data are relative to a construct lacking a mammalian promoter but containing no fragment from HER2. Statistics were calculated using a one-way ANOVA with significance represented as * *p* < 0.05, *** *p* < 0.005 and ns as not significant.

Having shown that HER2 CTFs are generated via internal initiation at several N-terminal methionine codons, we used a bicistronic reporter assay to investigate whether segments of the HER2 mRNA upstream of the CTFs could be responsible for initiating translation. We inserted 1 KB segments of HER2 RNA from the regions upstream of each functional AUG codon, encoding M611, M687 and M774 into the reporter downstream of a Renilla luciferase ORF and termination codon and immediately upstream of a firefly luciferase ORF (Figure 4a). To exclude a general effect of a long HER2 mRNA sequence inducing internal translation, we included two distinct 1 KB regions that were directly upstream of internal AUG codons, but which have no annotated protein products (pR347F and pR916F) as controls. RNA segments with significant capacity to initiate translation will yield increased expression of firefly relative to Renilla luciferase in cell lysates. All of the test regions (611, 687 and 774) have statistically significant increases in firefly activity compared to a no-insert control ranging from 8-fold to 39-fold. (Figure 4b and Figure S1). Neither of the control regions (347 and 916) have a significant increase in firefly activity compared to a no-insert control (Figure 4b). This ensures that the effect is specific to the regions of interest rather than just to having a 1 KB portion of the *HER2* mRNA. To exclude that the observed increases in activity were not due to a cryptic promoter, we introduced the same test sequences into a monocistronic firefly construct which lacks a mammalian promoter (Figure 4c). All constructs lacking a mammalian promoter had no luciferase activity, suggesting that no functional promoter is present in these regions (Figure 4d). This is in line with our data showing no expression of HER2 or HER2 CTFs from a HER2 expression construct which lacks a mammalian promoter (Figure 1b).

## 3. Discussion

Here, we show that at least three HER2 CTFs are generated by internal translation of the full *HER2* mRNA. Our use of in vitro-transcribed mRNA rules out the possibility of a cryptic promoter or splicing event, due to the stringent specificity of the T7 RNA polymerase and lack of splicing machinery in the in vitro transcription reaction. We were also able to rule out the possibility of a long-range scanning mechanism being responsible for HER2 CTF generation by blocking the cap binding and introducing a hairpin which prevents ribosomal scanning, both of which ablates the expression of the HER2 protein while reducing but sparing HER2 CTFs. The only remaining possibility is that the ribosome is binding internally on the *HER2* mRNA and initiating on a cluster of internal methionines to generate proteins.

This mechanism has been implicated for a number of other genes (reviewed in [23]. A few notable examples include *CACNA1A* [26], where a 220 KDa calcium channel is translated cap-dependently and a 75 KDa c-terminal protein is translated via an internal ribosome entry site (IRES); the internally translated protein has been shown to be critical for cerebellar development [27]. Critically, ectopic expression of a microRNA, which interacts with the IRES structure, has been shown to significantly reduce IRES function and expression of the 75 KDa c-terminal protein [28], further highlighting the importance of the mechanism and proving that it can be targeted by exogenous molecules for therapeutic gain. A viral form of *NOTCH2* has been shown to contain an IRES which promotes the expression of the NOTCH2 intracellular domain (NICD) and which drives gene expression changes. Interestingly, the region that was identified as a functional IRES is present in the cellular gene, suggesting that this may be a functional mechanism in mammalian cells [29]. Another example is *CDK11A*, where a p110 protein is expressed cap-dependently and a p58 protein is expressed via an internal IRES-like structure [30].

One other potential mechanism which could explain the generation of HER2 CTFs is ribosomal shunting. This mechanism is predominantly observed in plant cells and in viruses; however, one mammalian gene, *HSP70*, has also been shown to utilize ribosome shunting [31]. Ribosome shunting is a cap-dependent mechanism by which segments of an mRNA are skipped over by the ribosome, allowing initiation from downstream AUGs. As we have provided clear evidence that HER2 CTFs persist when cap-dependent translation is completely blocked by a 5′ hairpin structure and a non-functional A-cap, we do not believe that ribosome shunting is a valid mechanism to explain HER2 CTF generation.

Although critical previous studies have identified the start codons for the HER2 CTFs and shown important biological consequences of their associated proteins [11], none have unambiguously ruled out the possibility of a cryptic promoter or splicing event, or shown that regions of the *HER2* mRNA are able to induce internal translation in a reporter assay. Our data clearly rule out the possibility of a cryptic promoter and identify the important region required for internal translation. These data present regions of the *HER2* mRNA that may be ideal targets for development of ASO-based therapeutics to block internal initiation. Given the previous data on *CACNA1A*, in which ectopic expression of a microRNA is able to perturb IRES function [28], this is extremely important and represents a starting point for the development of this type of therapeutic for treatment-resistant HER2-amplified cancers.

Our data also shed light on the mechanism leading to internal translation of mRNAs. We clearly show that the presence of a functional cap structure, although not being required, is significantly beneficial for the translation of the HER2 CTFs (Figure 1d). mRNAs capped with non-functional cap structures are able to express HER2 CTFs, albeit at very low levels, suggesting that cap-dependent translation is not required, but is beneficial to internal translation. This may suggest that the internal translation may benefit from mRNA circularization or that a functional cap structure may bring into proximity any required initiation factors. Given that a hairpin structure at the 5′ of the mRNA did not significantly inhibit HER2 CTF generation, long-range scanning from the cap structure is unlikely to explain HER2 CTF generation. It is possible, however, that interactions between the region of RNA containing the internal start sites and the cap structure may occur, possibly through RNA binding proteins or non-coding RNAs. This possibility is worth investigating as it could identify proteins or RNAs that are vital for HER2 mRNA internal translation and might be important therapeutic targets. Being able to inhibit HER2 CTF expression while not impacting HER2 expression could resensitize resistant tumors to targeted therapeutics and offer important therapeutic opportunities.

## 4. Materials and Methods

All references to nucleotide positions are relative to *ERBB2* transcript variant 1 (NM_004448.4). All references to amino acid position are relative to receptor tyrosine-protein kinase ERBB-2 isoform A (NP_004439.2).

### 4.1. Cell Culture

HEK293T cells were purchased from ATCC. They were grown in DMEM supplemented with 10% FBS and 292 mg/mL L-glutamine in a humidified incubator at 37 °C in 10% CO_2_.

### 4.2. Mutagenesis

The wild-type HER2 FLAG-tagged expression plasmid was purchased from Addgene (16257). All subsequent mutagenesis and cloning were carried out on this plasmid. All mutagenesis carried out on this construct used the QuikChange II XL Site-Directed Mutagenesis Kit. The following primers were used to introduce all mutations used in this study, see Table 1:

### 4.3. In Vitro Transcription

In vitro transcription was achieved using the MEGAscript T7 In Vitro Transcription Kit (Invitrogen, Waltham, MA, USA) according to the manufacturer’s recommended protocol. In brief, plasmids were linearized with XBA1 and purified using the DNA Clean-Up Kit (Zymo Research, Irvine, CA, USA). The resulting linearized DNA was used as a template for in vitro transcription. For capped constructs, GTP was diluted 1:5, and 6 mM of either functional m^7^G(5′)ppp(5′)G RNA Cap Structure Analogue (NEB-S1404, Ipswich, MA, USA) or nonfunctional G(5′)ppp(5′)A RNA Cap Structure Analog (NEB-S1406, Ipswich, MA, USA) was used as a supplement. T7 Enzyme Mix was added at 1X and the reaction was incubated at 37 °C for 2 h. The reaction was then DNase-treated for 15 min at 37 °C.

For polyadenylated transcripts, the reaction was then supplemented with E-PAP buffer, 25 mM MnCl_2_ and 10 mM ATP, and poly(A)polymerase (PAP) before being incubated at 37 °C for 1 h. The resulting RNA was then column purified and stored at −80 °C until required.

### 4.4. Transfection

Transfection of DNA was achieved using Lipofectamine 2000 (Invitrogen, Waltham, MA, USA) according to the manufacturer’s instructions. In brief, cells were plated into wells of a 6-well dish and allowed to adhere overnight. A transfection mix containing 3 μL of Lipofectamine 2000 was used per 1 μg of plasmid DNA with a final volume of 200 μL. The reaction was incubated at room temperature for 20 min before being added dropwise to the cells. Cells were collected 48 h post-transfection.

Transfection of mRNA was achieved using TransIT-mRNA (Mirus Bio, Madison, WI, USA) according to the manufacturer’s instructions. In brief, 2.5 μg of mRNA was resuspended in 250 μL of Opti-MEM and mixed well. To this, 5 μL of the mRNA Boost Reagent and 5 μL of the TransIT mRNA Transfection Reagent were added and mixed well. The reaction was incubated at room temperature for 5 min before being added to cells. Cells were collected 24 h post-transfection.

### 4.5. SDS-PAGE

Cells were lysed in RIPA buffer (150 mM NaCl, 1% NP-40, 0.5% sodium deoxycholate, 0.1% SDS, 25 mM Tris pH 7.4) plus 1X Halt Protease Inhibitor Cocktail (Thermo Scientific, Waltham, MA, USA). Lysates were heat denatured in the presence of 1X Laemmli buffer and electrophoresed in a suitable percentage of acrylamide gel. Migrated proteins were transferred to a nitrocellulose membrane and blocked in 1X milk:TBST for an hour before being incubated with a suitable primary antibody overnight in 1X milk:TBST. Membranes were then washed 3X for 15 min in TBST before being incubated with a suitable secondary antibody in 1X milk:TBST for 2 h. Membranes were washed 3X with TBST before being incubated with Clarity Western ECL Substrate (Bio-Rad, Hercules, CA, USA) for 5 min and visualized using a ChemiDoc (Bio-Rad, Hercules, CA, USA). See Table 2.

### 4.6. Luciferase Assays

The 1000 bp regions directly upstream of the internally initiated methionines were cloned into dual-luciferase reporter plasmids using the SPE1 and NCO1 restriction sites between an upstream Renilla and a downstream firefly ORF using the following primers, see Table 3:

These plasmids were transfected into HEK293 cells for 24 h and *Renilla* and firefly activity were measured using the Dual-Luciferase Reporter System (Biorad-E1980 Hercules, CA, USA). Internal translation was reported as firefly activity normalized to *Renilla* activity.

The same regions of the HER2 mRNA were also cloned into a firefly luciferase reporter assay which lacks a mammalian promoter. These plasmids were co-transfected with a *Renilla* luciferase-expressing plasmid and activity was reported as firefly activity normalized to *Renilla* activity. The pRF plasmid was a gift from Dr. Spriggs KA.

### 4.7. Western Blot Quantification

Quantification of the relative intensity of protein bands on Western blots was achieved using the Image Lab 6.1.0 (Bio-Rad Hercules, CA, USA) quantity tool. Lanes and bands were automatically detected by the software and the adjusted band volumes were compared. The means and standard deviations from three biological repeats are reported.

### 4.8. Hairpin Construct Cloning

The following 5′ phosphorylated oligonucleotide was synthesized: AGCTTTCTGGTACCGAGCTCCCCGGGCtgcaGCCCGGGGAGCTCGGTACCAGAA. The oligonucleotide was diluted to 10 nM in annealing buffer (10 mM tris pH 7.5, 50 mM NaCl, 1 mM EDTA), heated to 95 °C and allowed to return to room temperature to allow duplexing. The HER2 plasmid was restricted at the HINDIII site and the duplexed oligo was ligated into the plasmid.

### 4.9. Kozak Context Analysis

The Netstart1.0 program [25] was used for analysis of the Kozak context of the HER2 mRNA. The entire HER2 transcript was analyzed for start codon prediction using default settings.

## Figures and Tables

**Table 1:** All primers used to generate constructs with stop codon insertions, frameshift mutations and start codon mutations.

Primer Name	Sequence
HER2 N68X Forward	cctacctgcccacctaagccagcctgtcctt
HER2 N68X Reverse	aaggacaggctggcttaggtgggcaggtagg
HER2 N360X Forward	gggcagttaccagtgcctaaatccaggagtttgctgg
HER2 N360X Reverse	ccagcaaactcctggatttaggcactggtaactgccc
HER2 N556X Forward	ccccagggagtatgtgtaagccaggcactgtttgc
HER2 N556X Reverse	gcaaacagtgcctggcttacacatactccctgggg
HER2 M611A Forward	ggaaacttccagatgggcgcgtaggagaggtcaggttt
HER2 M611A Reverse	aaacctgacctctcctacgcgcccatctggaagtttcc
HER2 M687A Forward	ctgcagcagtctccgcgccgtgtacttccggatc
HER2 M687A Reverse	gatccggaagtacacggcgcggagactgctgcag
HER2 M706A Forward	cgcctggttgggcgccgctccgctaggt
HER2 M706A Reverse	acctagcggagcggcgcccaaccaggcg
HER2 M712A Forward	tctttcaggatccgcgcctgcgcctggttggg
HER2 M712A Reverse	cccaaccaggcgcaggcgcggatcctgaaaga
HER2 M774A Forward	gagcccacaccagccgccacgtatgcttcgtc
HER2 M774A Reverse	gacgaagcatacgtggcggctggtgtgggctc
HER2 Frameshift 1 Forward	gaggattgtcagagctgacgcgcactgtct
HER2 Frameshift 1 Reverse	agacagtgcgcgtcagctctgacaatcctc
HER2 Frameshift 2 Forward	gaggattgtcagagcgacgcgcactgtctg
HER2 Frameshift 2 Reverse	cagacagtgcgcgtcgctctgacaatcctc
HER2 Frameshift 3 Forward	gaggattgtcagagcacgcgcactgtctgt
HER2 Frameshift 3 Reverse	acagacagtgcgcgtgctctgacaatcctc

**Table 2:** A list of all antibodies used in this study.

Target	Producer	Product Code	Species
FLAG (M2)	Sigma-Aldrich (St. Louis, MO, USA)	F1804	Mouse
GAPDH	Abcam (Cambridge, UK)	ab9485	Rabbit

**Table 3:** All primers used to generate bi-cistronic and mono citronic luciferase reporters.

Primer Name	Sequence
pR347F	atgcactagtcgcgagcacccaagtgtg
pR347F	atgcccatgggcccagaccatagcacactc
pR611F	atgcactagtcagccctggtcacctacaacacagac
pR611F	atgcccatgggtaggagaggtcaggtttcacaccgctg
pR687F	atgcactagtggcagttaccagtgccaatatc
pR687F	atgcccatggcgtgtacttccggatcttctgc
pR774F	atgcactagtctactcgctgaccctgcaagggctg
pR774F	atgcccatggcacgtatgcttcgtctaagatttctttgttggctttg
pR916F	atgcactagtctccacactgccaaccgg
pR916F	atgcccatggcagctcccacacagtcacac

## Data Availability

Not applicable.

## Supplementary Materials

The supporting information can be downloaded at: https://www.mdpi.com/article/10.3390/ijms23179549/s1.
