# Supplementary material for: HER2 c-Terminal Fragments Are Expressed via Internal Translation of the HER2 mRNA
Source: Int J Mol Sci. 2022 Aug 23;23(17):9549. doi: 10.3390/ijms23179549 (PMC9455161; doi:10.3390/ijms23179549)

Position	Score	Pred	Note	In Frame
150	0.528	Yes		FALSE
176	0.819	Yes	Full Length HER2 Start site	TRUE
266	0.767	Yes		TRUE
308	0.575	Yes		TRUE
378	0.212	-		FALSE
510	0.651	Yes		FALSE
531	0.629	Yes		FALSE
767	0.339	-		TRUE
885	0.623	Yes		FALSE
1019	0.449	-		TRUE
1152	0.288	-		FALSE
1203	0.133	-		FALSE
1214	0.538	Yes		TRUE
1320	0.335	-		FALSE
1417	0.609	Yes		FALSE
1488	0.631	Yes		FALSE
1804	0.43	-		FALSE
1836	0.286	-		FALSE
1842	0.396	-		FALSE
1887	0.604	Yes		FALSE
2006	0.744	Yes	M611 Start site	TRUE
2028	0.583	Yes		FALSE
2041	0.442	-		FALSE
2088	0.069	-		FALSE
2234	0.534	Yes	M687 Start site	TRUE
2291	0.46	-		TRUE
2309	0.514	Yes		TRUE
2400	0.242	-		FALSE
2409	0.171	-		FALSE
2495	0.45	-	M774 Start site	TRUE
2517	0.383	-		FALSE
2576	0.432	-		TRUE
2583	0.415	-		FALSE
2601	0.375	-		FALSE
2654	0.678	Yes		TRUE
2672	0.546	Yes		TRUE
2688	0.33	-		FALSE
2748	0.348	-		FALSE
2808	0.239	-		FALSE
2814	0.203	-		FALSE
2840	0.402	-		TRUE
2886	0.312	-		FALSE

2898	0.288	-		FALSE
2921	0.507	Yes		TRUE
2946	0.27	-		FALSE
3024	0.427	-		FALSE
3032	0.348	-		TRUE
3038	0.368	-		TRUE
3046	0.652	Yes		FALSE
3053	0.404	-		TRUE
3067	0.613	Yes		FALSE
3110	0.618	Yes		TRUE
3147	0.229	-		FALSE
3210	0.527	Yes		FALSE
3215	0.776	Yes		TRUE
3231	0.287	-		FALSE
3299	0.701	Yes		TRUE
3426	0.585	Yes		FALSE
3435	0.287	-		FALSE
3449	0.602	Yes		TRUE
3486	0.577	Yes		FALSE
3549	0.451	-		FALSE
3591	0.635	Yes		FALSE
3606	0.683	Yes		FALSE
3708	0.569	Yes		FALSE
3973	0.479	-		FALSE
4060	0.448	-		FALSE
4109	0.577	Yes		TRUE
4185	0.428	-		FALSE
4207	0.543	Yes		FALSE
4359	0.351	-		FALSE
4376	0.757	Yes		TRUE
4452	0.196	-		FALSE
4476	0.218	-		FALSE
4487	0.076	-		TRUE

Table S1. The output file from NetStart1.0 showing predicted start sites in the HER2 mRNA. Position represents the nucleotide position of the A in an AUG with potential to be a start site. Score represents the likeliness of an AUG being a functional start codon with scores greater than 0.5 being AUGs which are likely to be functional start codons. Pred is a boolean value with yes having a score greater than 0.5 and having a score less than 0.5. In frame as a boolean value showing if the AUG being interrogated is in frame with the canonical full length HER2 start site.

Supplemental Figure S1. Absolute luciferase activity of bicistronic luciferase reporters containing 1000 bp fragments upstream of the AUG codon encoding M611, M687 and M774, as well as two control constructs containing 1000 bp fragments of HER2 mRNA upstream of the AUG codons encoding M347 and M916.

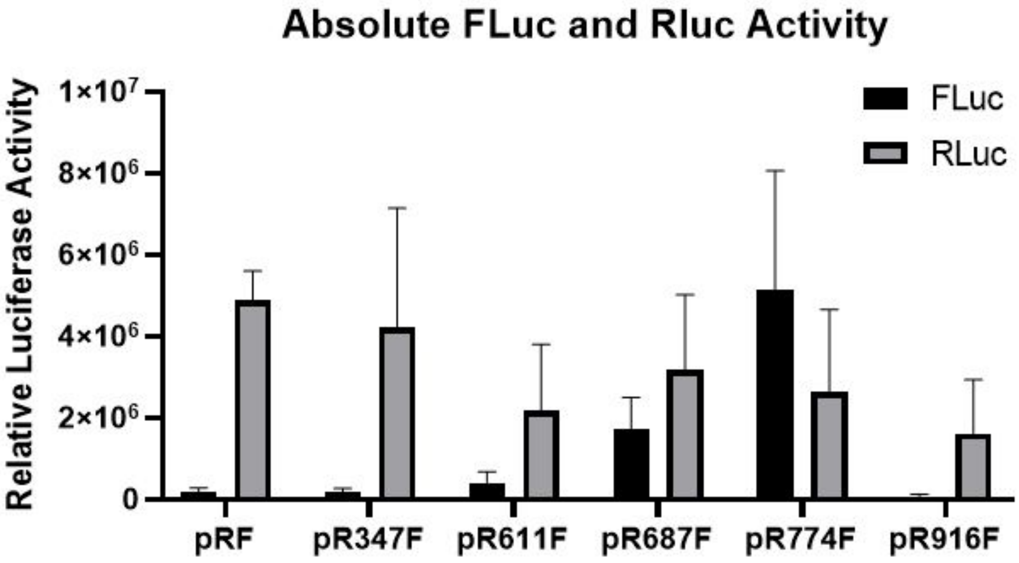

Supplement: Supplementary file 1 [file ijms-23-09549-s001.zip › ijms-1763797-supplementary.pdf]
